# Is There a Relationship between the Morphology of Connective Tissue and Reactivity during a Drop Jump? Influence of Sex and Athletic Performance Level

**DOI:** 10.3390/ijerph18041969

**Published:** 2021-02-18

**Authors:** Alberto Rubio-Peirotén, Felipe García-Pinillos, Diego Jaén-Carrillo, Antonio Cartón-Llorente, Luis E. Roche-Seruendo

**Affiliations:** 1 Campus Universitario, Universidad San Jorge, Autov A23 km 299, Villanueva de Gállego, 50830 Zaragoza, Spain; djaen@usj.es (D.J.-C.); acarton@usj.es (A.C.-L.); leroche@usj.es (L.E.R.-S.); 2 Department of Physical Education and Sports, Faculty of Sports Sciences, University of Granada, 18011 Granada, Spain; fegarpi@gmail.com; 3 Department of Physical Education, Sports and Recreation, Universidad de La Frontera, Temuco 4811230, Chile

**Keywords:** performance, running, tendon, ultrasound, jump reactivity

## Abstract

The influence of the morphologic characteristics of connective tissue, which plays an essential role during sports activities, on sporting tasks needs further research given the controversial findings reported in the literature. This study aimed at examining the relationship between lower limb connective tissue morphology and drop jump reactivity and determining the influence of sex and athletic performance level. A total of 30 men and 14 women, runners, executed 6 drop jumps (3 × 20 cm and 30 cm height respectively) and their thickness and cross-sectional area were recorded for Achilles and patellar tendons and plantar fascia. No significant results were found in the relationship between the morphology of the connective tissue and reactivity strength index for both sexes. Significant sex differences were found, while women showed greater values (*p* < 0.005) for Achilles tendon and plantar fascia; men showed greater values for reactivity strength index and drop jump performance (*p* < 0.001). The present study shows a limited relationship between connective tissue morphology and drop jump reactivity. Additionally, women showed greater normalized values for Achilles tendon and plantar fascia, and men showed greater reactivity strength index and jumping performance values. No relationships between athletic performance level and connective tissue were found.

## 1. Introduction

The relationship between the anatomic structure and its function has been widely investigated. Determining whether the structure dictates the function, or this cause–consequence relation follows a bidirectional way is supported by previous work [1,2].

Given the determinant role of the connective tissue within this structure, especially the tendon, its influence on sports activities such as running and jumping has been examined previously [1,3]. It is suggested that tendon properties such as stiffness, thickness and cross-sectional area (CSA) constitute an important part of such sports tasks [4]. Monte et al. [4] showed that Achilles tendon (AT) CSA and length positively correlated with sprint running performance (with values of velocity, force and power). However, at the same time, the mechanical use influences the adaptation of that tendon. It has been shown that higher workloads can modify tendon stiffness reducing the effect caused by aging [2]. Therefore, regarding the structure–function relation, it seems that both structure and function can behave as cause and consequence.

One reason that might explain why the tendon is the spotlight of the study of the structure–function relationship is the process of energy transformation. It is well known the outstanding importance of the tendon structure during the process of store–release energy, which allows the reduction of metabolic energy used and consequently increases the economy of sporting tasks such as running or jumping [5].

The stretch-shortening cycle (SSC) is one of the main neuromuscular mechanisms for such store–release energy process [6]. The SSC is characterized by a lengthening phase where the muscle is acting eccentrically followed by a concentric (shortening) phase [7]. The leg behaves like a spring which compresses and decompresses continually during the SSC [8,9]. Mechanical energy is stored by the muscle-tendon units over the leg-spring compression, represented by the eccentric phase of stance, whereas the concentric phase of stance releases part of the elastic energy stored [7]. Nevertheless, it has been proposed that without tendons, the muscle shortening speed during fast movements such as jumps would increase; consequently, the energy storage would decrease resulting in a lower outcome for a particular task (i.e., jumping) [5].

The reactivity strength index (RSI), which refers to the ability to switch rapidly from an eccentric to a concentric contraction, has supported its efficacy while measuring body explosive performance [10]. The RSI can be calculated from the ratio of flight time (FT) and contact time (CT) when performing a drop jump (DJ) [11]. DJ is required “to jump as high and as fast as possible” after landing, which is quite similar to plyometric exercises [12]. In this way, the RSI can be used to assess the performance of plyometric exercises [13]. It has been previously shown that some factors (i.e., sex) influence RSI, showing men greater values than women over different sports [14,15]. 

Moreover, sex differences are also present in the connective tissue morphology. Specifically, males demonstrated significantly larger AT-CSA than females [16]. This finding must be considered cautiously as, in many studies such as the one cited, tendon values were not normalized in relation to body mass, since if the samples are not homogeneous in relation to the body mass, the results after normalizing the tendon values in relation to the body mass might be different [3]. The influence of body mass should be considered when studying tendon properties, as it has been previously reported that subjects with lower body mass index exhibit lower AT-CSA [17]. 

Another factor that can relate to the morphologic characteristics of the connective tissue is the athletic performance level. Previous studies demonstrated that runners have a greater AT-CSA compared with sedentary or mildly active individuals [18,19]. However, to the authors’ knowledge, this comparison has not been conducted in a homogeneous sample of amateur endurance runners considering the influence of the athletic performance level and sex difference, assessing the main tendons of the lower limb.

Based on the current literature, how sex and athletic performance level influence both connective tissue morphology and RSI seems to remain unclear. Therefore, the aim of this study is twofold: (i) to examine the relationship between the morphology of patellar tendon (PT), AT and plantar fascia (PF), and RSI during a DJ in amateur endurance runners; and (ii) to determine the influence of sex and athletic performance level on the morphology of the aforementioned structures and RSI during a DJ. Taking into account that previous studies [15,16] found that men showed both higher values of RSI and AT-CSA, we hypothesized that greater values of the connective tissue characteristics exhibit a greater RSI, and the variables of sex and athletic performance level influence this relationship. Additionally, given the differences in connective tissue in absolute values mentioned above, we also hypothesized that men would show greater values for normalized tendon thickness and CSA than women. 

## 2. Materials and Methods

### Subjects

A total of 44—30 men and 14 women—amateur endurance runners voluntarily participated in this study (age: 28.1 ± 6.9 years; height: 172.3 ± 7.7 cm; body mass: 67.3 ± 9.9 kg; BMI: 22.6 ± 2.2, body fat %: 15.21 ± 4.98; lean mass % 80.88 ± 5.75). All participants met the inclusion criteria: [i] older than 18 years old, [ii] three or more training sessions per week [20], and [iii] not suffering from any injury in the last 6 months before the data collection. After receiving detailed information on the objectives and procedures of the study, each participant signed an informed consent form in order to participate, which complied with the ethical standards of the World Medical Association’s Declaration of Helsinki (2013). It was made clear that the participants were free to leave the study if they saw fit. The study was approved by the Institutional Review Board (006-18/19).

## 3. Procedures

Data were collected over only one session in a biomechanics laboratory during March and April 2019. Every subject performed exactly the same procedure, instructed and supervised by a researcher. Regarding athletic performance level, the subjects were assigned to the higher-level group (HLG) and lower-level group (LLG). 

A warm-up protocol was developed by each participant before the start of the testing session. It consisted of 5-min stationary cycling, dynamic stretching consisting of leg CMJ with bounce, and ankle jumps. It is suggested that this type of warm-up stimulates greater jumping performance [21]. Each subject performed 3 maximal jumps at each of the two DJ heights (20 [DJ20] and 30 cm [DJ30], respectively) and the highest jump from each height was taken for the subsequent analysis. The landing zone was established between two transmitting-receiving bars belonging to a photoelectric cell system (OptoGait, Microgate, Bolzano, Italy)—previously validated to measure vertical jump height [22]. Measurements of FT (ms) and CT (ms) were recorded, and their ratio for both heights (RSI20 and RSI30) was found. The RSI reliability and validity were reported to measure body explosive performance [10,14]. Participants had a 1-min rest between jumps and a 3-min recovery between DJ heights [23]. To start, participants were asked to “step out” from the box, keeping their hands on their hips to reduce arm movement, and “to jump as high and as fast as possible” after landing [24]. Jumps were considered unacceptable when the participants’ legs were not extended over the flight or they jumped off the landing zone. 

## 4. Materials and Testing

As participants entered the laboratory, body mass (kg) and height (cm) were determined using a weighing scale (Tanita BC-601; TANITA Corp., Maeno-Cho, Itabashi-ku, Tokyo, Japan) and a stadiometer (SECA 222; SECA Corp., Hamburg, Germany) for descriptive purposes. 

### 4.1. RSI

In order to calculate RSI, CT (time the foot spends in contact with the ground) and FT (time from toes off to the next contact) during the DJ performance were recorded. For this purpose, the OptoGait system was placed as explained above and was linked to a laptop being the manufacturer software used (Version 1.12.1.0, Microgate, Bolzano, Italy). Furthermore, data were collected at a sampling frequency of 1000 Hz. 

### 4.2. Ultrasound Measurements

An ultrasonic device (LOGIQ S7 EXPERT, General Electric, Germany, 2013) with an electronic linear array probe (ML 6–15 MHz. MATRIX LINEAR) was used to obtain B-mode ultrasonic images of AT, PT and PF.

To perform the ultrasound (US) assessment of the PT, the subject was placed on a stretcher in a supine position with both knees bent at 30° [25]. The thickness of the tendon was measured with the probe placed longitudinally at the reference of 1 cm distal to the lower pole of the patella. This point was identified using a skin mark. At the same point, the CSA was measured placing the probe in a transversal way. The images were taken with a depth of 3 cm and the focus at 0.5 cm.

To perform the US assessment of the AT, the subject was placed in a prone position, with the ankle in a neutral position with the feet hanging outside of the stretcher [25]. The thickness of the tendon was measured with the probe placed longitudinally at the reference of 3 cm proximal to the insertion of the tendon in the calcaneus bone. This point was also identified using a skin mark. At the same point, CSA was measured placing the probe in a transversal way. The images were taken with a depth of 2cm and the focus at 0.5 cm. 

Then the PF was also assessed keeping the subject in the same position with the ankle in a neutral position and fingers extended against the stretcher surface [25]. The PF thickness was measured with the probe placed longitudinally at the reference located from the anterior edge of the plantar surface of the calcaneus bone vertically to the anterior edge of the PF. This point was identified using a skin mark.

All the images were taken with a frequency of 12 Mhz and gain of 100 dB. Each measurement was recorded twice by a skilled researcher with more than ten years of experience in diagnostic ultrasound imaging. Before the statistical analysis, thickness and CSA of every tendon were measured using the software ImageJ (NIH, Baltimore, MD, USA) [26]. Due to the close relationship found previously between body mass and tendon morphology characteristics [27], the CSA and thickness values were normalized to one-third of the body mass [1].

## 5. Statistical Analysis 

Descriptive data are presented as mean and standard deviation (± SD), while nominal variables are presented as frequency and percentage (*n*, %). The normality distribution of the data was confirmed by Shapiro–Wilk’s test (*p* > 0.05). To determine the intra-rater reliability of the measures related to the morphology of the connective tissue, intraclass correlation coefficients (ICCs) were calculated for each parameter. Additionally, the 95% confidence interval (CI) of the ICC value was provided [28]. In order to analyze the relationship between tissue morphology parameters and jumping performance parameters, a Pearson correlation analysis was conducted for each sex. The following criteria were adopted to interpret the magnitude of correlations between measurement variables: <0.1 (trivial), 0.1–0.3 (small), 0.3–0.5 (moderate), 0.5–0.7 (large), 0.7–0.9 (very large) and 0.9–1.0 (almost perfect) [29]. An analysis of variance (ANOVA) was conducted between sexes for each dependent variable (i.e., connective tissue morphology and jumping performance parameters). Additionally, a cluster k-means analysis was conducted regarding the athletic performance level in terms of 10 km personal best, to split the whole-group into a higher-level group (HLG) and lower-level group (LLG), and an ANCOVA was conducted between level groups, considering the sex as a covariate. The Chi2 test was used to compare the sex distribution between BMI groups. Finally, the magnitude of the differences between values was also interpreted using Cohen’s d effect size (*ES*) (between-group differences) [30]. Effect sizes are reported as: trivial (<0.19), small (0.2–0.49), medium (0.5–0.79), and large (≥0.8) (Cohen, 1988). All statistical analyses were performed using SPSS software version 25.0 (SPSS Inc., Chicago, IL, USA) and statistical significance was accepted at an alpha level of 0.05.

## 6. Results

Given that previous studies suggest differences by sex in the variables analyzed [15,16], the correlation analysis was conducted independently for each sex (Table 1). For men, the Pearson correlation analysis reported an inverse significant correlation between normalized PT-CSA with RSI30 (r = −0.475, *p* < 0.01). For women, the analysis reported no significant correlation between the connective tissue and the RSI20 and RSI30 (*p* ≥ 0.05).

Excellent intra-rater reliability was reported for the measures related to the morphology of the connective tissue (ICC > 0.989, 95% CI: 0.913–0.996).

**Figure 1:**
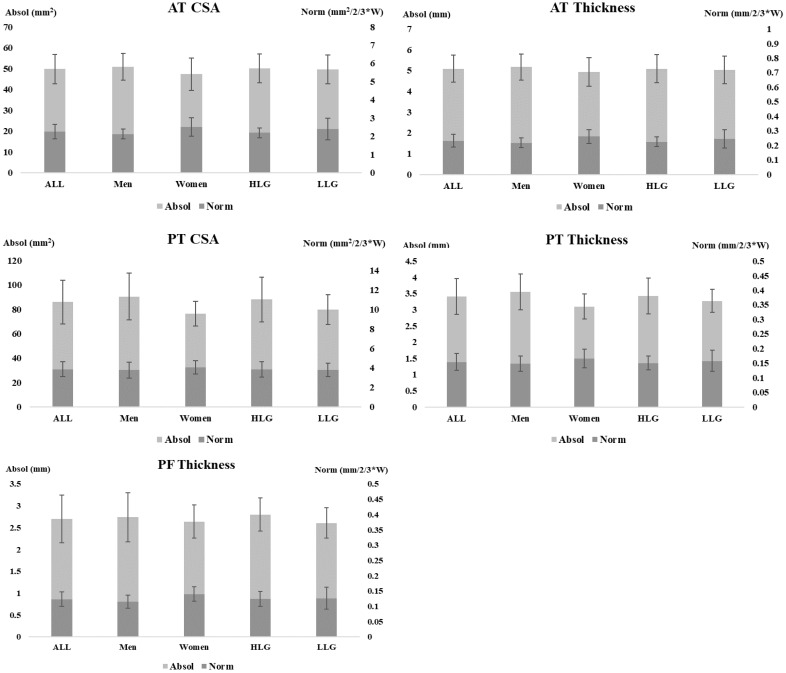
Comparative between normalized to body mass values vs. absolute values for connective tissue. Comparative between normalized to body mass values vs. absolute values for connective tissue. ALL: Full sample; Absol: Connective tissue absolute values; Norm: Connective tissue normalized values; PT: Patellar tendon; AT: Achilles tendon; PF: Plantar fascia; CSA: Cross-sectional area; HLG: higher athletic-level group; LLG: lower athletic-level group. W: Weight.

A comparison between the absolute values and the normalized values for the body mass of the connective tissue is shown (Figure 1). These data appear both for the complete sample and for the comparison between sexes and athletic performance level.

The comparison between sex showed greater values for women in all parameters related to the morphology of the connective tissue (Table 2), though significant between-sex differences were found in normalized AT-Thickness, normalized AT-CSA, and normalized PF-Thickness (*p* < 0.01, ES = 0.6). No significant differences between sexes were found in the normalized PT-Thickness and normalized PT-CSA (*p* ≥ 0.05). Greater values were reported in men for DJ performance parameters (i.e., DJ20, DJ30, RSI20 and RSI30), with significant between-sex differences in all aforementioned parameters (*p* ≤ 0.001, ES > 0.8).

The cluster analysis split the whole group regarding athletic performance level into HLG (*n* = 24, 18 men and 6 women, 10 km time trial= 42.8 ± 3.5) and LLG (*n* = 20, 9 men and 11 women, 10 km time trial = 52.3 ± 3.3) (*p* < 0.001). The sex distribution showed significant between-group differences (Chi2 = 0.017) so, sex was considered as a covariate. Table 3 shows the morphology of the connective tissue and DJ performance parameters regarding athletic performance level. The ANCOVA, adjusted by sex, revealed no between-group differences in the morphology of the connective tissue, but significant differences between level groups in DJ performance parameters with the HLG reporting better jumping performance. 

## 7. Discussion

This study aimed at assessing the relationship between the morphology of the connective tissue and the reactivity during a DJ as well as the sex and athletic performance level influence in amateur endurance runners. The major finding of this study was the there is no significant relationship between the morphology of the connective tissues (i.e., PT, AT and PF, in terms of normalized thickness and normalized CSA) and RSI. However, a significant negative correlation for men was found between PT-CSA and RSI30. Regarding the influencing factors, women showed greater values of AT-Thickness, AT-CSA and PF-Thickness when normalized to body mass. On the other hand, men obtained greater values of height and RSI for both DJ heights. Additionally, no relationship was found for the athletic performance level. 

Our results support the findings by Earp et al. [31] where no relation between AT and PT thickness with DJ performance was found. Although only trained participants were included in both studies, Earp et al. [31] only considered men, omitting the possible sex differences in such tasks. Murtagh et al. [3] found no relation between PT-CSA and vertical jump in elite football players. This study used unilateral countermovement jumps (CMJ) to assess the vertical jump performance, whereas bilateral DJ was used in our study. As mentioned above, a significant negative correlation between PT-CSA and RSI30 (r = −0.475) was found for men. This finding can be supported by the specificity principle of the task. As explained in the methods section, the DJ seeks the lowest ground contact time, explaining that the AT is mainly required (over PT). Similarly, the CMJ looks for the highest height, demanding a greater implication of the quadriceps muscle and therefore the PT. Maybe for this reason Murtagh et al. [3] show a trend towards a positive correlation between the PT-CSA and CMJ, although the results were not significant. Contrary to the studies mentioned above, Zellers et al. [32] found that after 12 months of AT rupture, the AT-CSA had a positive relation with vertical jump performance. This study assesses jump performance with CMJ and drop CMJ (i.e., unilateral DJ followed by a vertical jump on one leg). It is worth mentioning that Zellers and colleagues [32] considered only participants with a prior AT rupture. It seems clear that the degree of the load to which the tendon had been subjected was very different between both samples since it has been shown that workload influences tendon properties [27]. It is well known that tendon stiffness, highly influenced by load, instigates a positive effect on vertical jump performance [33]. Consequently, it is arguable that in tendons under a low level of load after a rupture, other parameters such as tendon stiffness appear to be less developed. Under these circumstances, morphological characteristics of connective tissue (i.e., CSA and thickness) may relate to jump performance. 

Despite the lack of significance, the results of our study show a trend in a positive correlation between the characteristics of the AT and the PF with respect to the RSI. These findings can be explained regarding the characteristics of the DJ. In order to perform a DJ properly, the subject has “to jump as high and as fast as possible” [24], trying to minimize ground contact time over the task; as explained above, the lower limb behaves such as a spring [8,9], facilitating the SSC mechanism in the AT and PF during the DJ. Of note, the decreased knee flexion shown during the DJ prevents the SCC from acting in the PT, which could explain the negative correlation between the PT characteristics and the RSI for both heights. It seems to be that, in relation to the demands that the activity provokes in the tendon, the morphological characteristics of these tendons tend to correlate positively with the performance of such activity. Likewise, activities such as plyometric exercises, quite like the DJ, that look for the greatest reactivity could involve mainly the AT and the PF, whereas other types of activities looking for power and height in the jump (i.e., basketball, volleyball) would demand greater implication of the quadriceps muscle and the PT.

The current study also analyzed the influence of both sex and athletic performance level on the morphology of PT, AT, and PF and the RSI of the lower limb. 

In relation to sex influence, the results obtained show the existence of between-sex differences in the morphology of the connective tissue (i.e., women obtained greater normalized to body mass values of AT-Thickness, AT-CSA and PF-Thickness) and jump performance parameters (i.e., men obtained greater values of height and RSI for both DJ heights). Opposite to our preliminary hypothesis, women showed greater values of AT-Thickness, AT-CSA and PF-Thickness, than men when normalized to body mass. The results were the opposite when absolute thickness and CSA values were compared showing men greater values than women. This finding highlights the importance of normalizing tendon thickness and CSA to the body mass, when these characteristics are to be correlated with other variables (i.e., RSI). As explained in the methods section, there is a clear influence of body mass on the morphologic characteristics of the tendon [27] and, thereby, it is important to consider the normalized values [18,34], especially when the sample is heterogeneous, the results might be reversed [3] as it occurs in the present study. 

A recent study [17] examined sex differences related to AT thickness and CSA. The authors reported opposite findings to the current study. Kudron and colleagues [17] showed that men had greater AT-CSA than women. However, Kudron and colleagues used absolute values of AT-CSA. It remains unknown whether after normalizing the connective tissue characteristics to body mass, the results would continue in the same direction or, instead, women would show greater values as the sample used were runners with a similar age to our study but different performance level (the study assessed Division I collegiate cross-country runners). 

In the case of the athletic performance level influence, a lack of influence on the morphology of the connective tissue was found (*p* ≥ 0.164 (0.38) for all the connective tissue values). These findings are in disagreement with previous studies [18,19]. Those studies showed a greater AT-CSA in long-distance runners compared to the control group. A possible explanation to this discrepancy might be the difference in the athletic performance level between both groups. In the current study, all the participants were runners and, to compare in relation to athletic performance level, the sample was divided according to their best 10k time. It is worth highlighting that other studies [19] did not consider runners for their control group and, in some cases, the differences between the running group (i.e., best 5km time: 14.43 ± 0.16 min) and the control group were huge [18]. Probably this level of moderate physical activity, such as in our sample, can act as prophylaxis to avoid thinning of the tendon. Again, it is essential to underline the relationship between the load to which the tendon is subjected and its morphological characteristics.

Continuing with the athletic performance level, HLG subjects showed a better jump performance but the differences were not significant. In light of the results discussed above, other factors (i.e., muscle characteristics), different from the normalized tendon thickness and normalized CSA, would be the responsibility of these findings.

Despite the findings reported here, there are some limitations to be considered. Only amateur endurance runners were considered for the study, remaining unknown the outcomes for higher-level runners or sedentary, as well as their likely differences. This limitation of the sample means that its characteristics are very specific to the study population, which may imply a bias, however, this limitation also allows the results obtained to be more easily interpreted due to the greater homogeneity of the sample. Another limitation to take into account is that we studied the sex influence, but both menstrual cycle phases and contraceptive intake were controlled, preventing the study from clarifying how they may affect the connective tissue. Sex differences in connective tissue characteristics need further research since it is unclear whether training adaptation or hormonal disturbance determines sex differences [35]. Notwithstanding these limitations, the current study examines the relationship between the connective tissue characteristics and the RSI during the DJ and highlights the influence of sex and athletic level. 

## 8. Conclusions

Although there is no significant relationship between the morphology of the connective tissue and jump performance in amateur endurance runners, the present study reveals a trend towards a positive correlation between the morphology of the AT and the PF with respect to the RSI. Additionally, this study highlights the potential influence of some parameters; whereas the athletic performance level seems not to mediate the morphology of the tendons analyzed, between-sex differences were found in the morphology of connective tissue, showing women greater values for AT-Thickness, AT-CSA and PF-Thickness when normalized to body mass. 

Moreover, morphologic values should be normalized in relation to the body mass, especially when using heterogeneous samples. Besides, sex differences should be considered.

From a practical standpoint, it seems that the characteristics of the task can determine the relationship between the connective tissue and the activity performance. Thus, in activities that involve repetitive rebounds such as plyometric exercises, where the objective is to obtain the shortest contact time, greater AT and PF could be essential. 

## Figures and Tables

**Table 1:** Relationship (coefficient r) between normalized to body mass connective tissue morphology and jumping performance parameters.

**Men (*n* = 30)**		
	RSI20	RSI30
PT-thickness	−0.050	−0.233
PT-CSA	−0.203	−0.475 *
AT-thickness	−0.053	−0.114
AT-CSA	0.111	0.079
PF-thickness	0.140	0.160
**Women (*n* = 14)**		
	RSI20	RSI30
PT-thickness	−0.240	−0.224
PT-CSA	−0.373	−0.300
AT-thickness	0.143	0.080
AT-CSA	0.395	0.283
PF-thickness	0.375	0.421

Note: * *p* < 0.01. PT: Patellar tendon; AT: Achilles tendon; PF: Plantar fascia; CSA: Cross-sectional area; RSI: reactive strength index.

**Table 2:** Morphology of the normalized to body mass connective tissue and drop jump performance parameters regarding sex.

Variable	All (*n* = 44)	Sex		*p*-Value (ES)	ES
		Men (*n* = 30)	Women (*n* = 14)		
Connective tissue					
PT-thickness	0.154 (0.029)	0.149 (0.026)	0.166 (0.032)	0.065 (0.62)	0.58
PT-CSA	3.869 (0.772)	3.782 (0.805)	4.054 (0.687)	0.282 (0.59)	0.36
AT-thickness	0.232 (0.043)	0.218 (0.034)	0.261 (0.047)	0.001 (1.09)	1.04
AT-CSA	2.253 (0.397)	2.132 (0.260)	2.511 (0.513)	0.002 (1.16)	0.93
PF-thickness	0.123 (0.024)	0.115 (0.022)	0.140 (0.025)	0.001 (0.87)	1.06
Drop jump performance parameters					
DJ20 (cm)	24.25 (5.85)	26.26 (5.72)	20.09 (3.48)	<0.001 (1.24)	1.30
RSI20	1.96 (0.52)	2.13 (0.48)	1.59 (0.41)	<0.001 (1.17)	1.21
DJ30 (cm)	26.07 (6.30)	28.25 (6.23)	21.57 (3.48)	<0.001 (1.26)	1.32
RSI30	2.11 (0.53)	2.29 (0.47)	1.74 (0.46)	<0.001 (1.38)	1.18

Note: ES: Cohen’s d effect size; PT: Patellar tendon; AT: Achilles tendon; PF: Plantar fascia; CSA: Cross-sectional area; DJ20: Drop jump from a 20 cm box; RSI: reactive strength index; DJ30: Drop jump from a 30 cm box.

**Table 3:** Morphology of the normalized to body mass connective tissue and drop jump performance parameters regarding athletic performance level.

Variable	All (*n* = 44)	Athletic Performance		*p*-Value (ES)	ES
		HLG (*n* = 24)	LLG (*n* = 20)		
Connective tissue					
PT-thickness	0.154 (0.029)	0.151 (0.024)	0.158 (0.036)	0.853 (0.62)	0.23
PT-CSA	3.869 (0.772)	3.866 (0.793)	3.800 (0.700)	0.342 (0.67)	0.09
AT-thickness	0.232 (0.043)	0.224 (0.033)	0.244 (0.064)	0.909 (0.31)	0.39
AT-CSA	2.253 (0.397)	2.198 (0.265)	2.392 (0.588)	0.834 (0.55)	0.43
PF-thickness	0.123 (0.024)	0.124 (0.022)	0.126 (0.024)	0.164 (0.38)	0.09
Drop jump performance parameters					
DJ20 (cm)	24.25 (5.85)	24.09 (5.66)	23.07 (5.63)	0.547 (0.16)	0.18
RSI20	1.96 (0.52)	2.04 (0.49)	1.74 (0.49)	0.288 (0.41)	0.61
DJ30 (cm)	26.07 (6.30)	25.91 (5.91)	24.68 (5.67)	0.592 (0.24)	0.21
RSI30	2.11 (0.53)	2.21 (0.56)	1.93 (0.48)	0.465 (0.33)	0.54

Note: HLG: higher-level group; LLG: lower-level group; ES: Cohen´s d effect size; PT: Patellar tendon; AT: Achilles tendon; PF: Plantar fascia; CSA: Cross-sectional area; DJ20: Drop jump from a 20 cm box; DJ30: Drop jump from a 30 cm box; RSI: reactive strength index. ^ Analysis of covariance with sex as covariate.

## Data Availability

The data presented in this study are available on request from the corresponding author. The data are not publicly available due to authors preferences.

## References

[1] Kubo K., Tabata T., Ikebukuro T., Igarashi K., Yata H., Tsunoda N. (2010). Effects of mechanical properties of muscle and tendon on performance in long distance runners. Eur. J. Appl. Physiol..

[2] Reeves N.D. (2006). Adaptation of the tendon to mechanical usage. J. Musculoskelet. Neuronal Interact..

[3] Murtagh C.F., Stubbs M., Vanrenterghem J., O’Boyle A., Morgans R., Drust B., Erskine R.M. (2018). Patellar tendon properties distinguish elite from non-elite soccer players and are related to peak horizontal but not vertical power. Eur. J. Appl. Physiol..

[4] Monte A., Zamparo P. (2019). Correlations between muscle-tendon parameters and acceleration ability in 20 m sprints. PLoS ONE.

[5] Gruber M., Kramer A., Mulder E., Rittweger J. (2019). The Importance of Impact Loading and the Stretch Shortening Cycle for Spaceflight Countermeasures. Front. Physiol..

[6] Vogt M., Hoppeler H.H. (2014). Eccentric exercise: Mechanisms and effects when used as training regime or training adjunct. J. Appl. Physiol..

[7] Komi P.V. (2000). Stretch-shortening cycle: A powerful model to study normal and fatigued muscle. J. Biomech..

[8] Blickhan R. (1989). The spring-mass model for running and hopping. J. Biomech..

[9] McMahon T.A., Cheng G.C. (1990). The mechanics of running: How does stiffness couple with speed?. J. Biomech..

[10] Kipp K., Kiely M.T., Geiser C.F. (2016). Reactive Strength Index Modified Is a Valid Measure of Explosiveness in Collegiate Female Volleyball Players. J. Strength Cond. Res..

[11] Beattie K., Carson B.P., Lyons M., Kenny I.C. (2017). The relationship between maximal strength and reactive strength. Int. J. Sports Physiol. Perform..

[12] Davies G., Riemann B.L., Manske R. (2015). Current Concepts of Plyometric Exercise. Int. J. Sports Phys. Ther..

[13] Snyder B.W., Munford S.N., Connaboy C., Lamont H.S., Davis S.E., Moir G.L. (2018). Assessing Plyometric Ability during Vertical Jumps Performed by Adults and Adolescents. Sports.

[14] Suchomel T.J., Sole C.J., Bailey C.A., Grazer J.L., Beckham G.K. (2015). A comparison of reactive strength index-modified between six US collegiate athletic teams. J. Strength Cond. Res..

[15] Sole C., Suchomel T., Stone M. (2018). Preliminary Scale of Reference Values for Evaluating Reactive Strength Index-Modified in Male and Female NCAA Division I Athletes. Sports.

[16] Intziegianni K., Cassel M., Hain G., Mayer F. (2017). Gender Differences of Achilles tendon Cross-sectional Area during Loading. Sports Med. Int. Open.

[17] Kudron C., Carlson M.J., Meron A., Sridhar B., Brakke Holman R. (2020). Using Ultrasound Measurement of the Achilles Tendon in Asymptomatic Runners to Assist in Predicting Tendinopathy. J. Ultrasound Med..

[18] Kubo K., Miyazaki D., Yamada K., Yata H., Shimoju S., Tsunoda N. (2015). Passive and active muscle stiffness in plantar flexors of long distance runners. J. Biomech..

[19] Magnusson S.P., Kjaer M. (2003). Region-specific differences in Achilles tendon cross-sectional area in runners and non-runners. Eur. J. Appl. Physiol..

[20] Piercy K.L., Troiano R.P., Ballard R.M., Carlson S.A., Fulton J.E., Galuska D.A., George S.M., Olson R.D. (2018). The Physical Activity Guidelines for Americans. JAMA.

[21] Cormack S.J., Newton R.U., McGuigan M.R., Doyle T.L. (2008). Reliability of measures obtained during single and repeated countermovement jumps. Int. J. Sports Physiol. Perform..

[22] Glatthorn J.F., Gouge S., Nussbaumer S., Stauffacher S., Impellizzeri F.M., Maffiuletti N.A. (2011). Validity and reliability of Optojump photoelectric cells for estimating vertical jump height. J. Strength Cond. Res..

[23] Markwick W.J., Bird S.P., Tufano J.J., Seitz L.B., Haff G.G. (2015). The intraday reliability of the reactive strength index calculated from a drop jump in professional men’s basketball. Int. J. Sports Physiol. Perform..

[24] Young W.B., Pryor J.F., Wilson G.J. (1995). Countermovement and Drop Jump Performance. J. Strength Cond. Res..

[25] Del Bano-Aledo M.E., Martinez-Paya J.J., Rios-Diaz J., Mejias-Suarez S., Serrano-Carmona S., de Groot-Ferrando A. (2017). Ultrasound measures of tendon thickness: Intra-rater, Inter-rater and Inter-machine reliability. Muscles Ligaments Tendons J..

[26] Kernozek T.W., Knaus A., Rademaker T., Almonroeder T.G. (2018). The effects of habitual foot strike patterns on Achilles tendon loading in female runners. Gait Posture.

[27] Rosager S., Aagaard P., Dyhre-Poulsen P., Neergaard K., Kjaer M., Magnusson S.P. (2002). Load-displacement properties of the human triceps surae aponeurosis and tendon in runners and non-runners. Scand. J. Med. Sci. Sports.

[28] Koo T.K., Li M.Y. (2016). A Guideline of Selecting and Reporting Intraclass Correlation Coefficients for Reliability Research. J. Chiropr. Med..

[29] Hopkins W.G., Marshall S.W., Batterham A.M., Hanin J. (2009). Progressive statistics for studies in sports medicine and exercise science. Med. Sci. Sports Exerc..

[30] Cohen J. (1988). Statistical Power Analysis for the Behavioral Sciences.

[31] Earp J.E., Kraemer W.J., Cormie P., Volek J.S., Maresh C.M., Joseph M., Newton R.U. (2011). Influence of muscle-tendon unit structure on rate of force development during the squat, countermovement, and drop jumps. J. Strength Cond Res..

[32] Zellers J.A., Pohlig R.T., Cortes D.H., Gravare Silbernagel K. (2020). Achilles tendon cross-sectional area at 12 weeks post-rupture relates to 1-year heel-rise height. Knee Surg. Sports Traumatol. Arthrosc..

[33] Kubo K., Kawakami Y., Fukunaga T. (1999). Influence of elastic properties of tendon structures on jump performance in humans. J. Appl. Physiol..

[34] Kongsgaard M., Aagaard P., Kjaer M., Magnusson S.P. (2005). Structural Achilles tendon properties in athletes subjected to different exercise modes and in Achilles tendon rupture patients. J. Appl. Physiol..

[35] Hansen M., Kjaer M. (2016). Sex Hormones and Tendon. Adv. Exp. Med. Biol..

